# Early sexual maturation, central adiposity and subsequent overweight in late adolescence. A four-year follow-up of 1605 adolescent Norwegian boys and girls: the Young HUNT study

**DOI:** 10.1186/1471-2458-7-54

**Published:** 2007-04-12

**Authors:** Grete H Bratberg, Tom IL Nilsen, Turid L Holmen, Lars J Vatten

**Affiliations:** 1 HUNT Research Centre, Faculty of Medicine, Norwegian University of Science and Technology, Verdal, Norway; 2 Department of Public Health and General Practice, Faculty of Medicine, Norwegian University of Science and Technology, Trondheim, Norway; 3 Nord-Trøndelag University College, Levanger, Norway

## Abstract

**Background:**

Early sexual maturation has been associated with overweight that may persist after the completion of biological growth and development. We have prospectively examined the influence of early sexual maturation on subsequent overweight in late adolescence and assessed if this association was modified by central adiposity in early adolescence.

**Methods:**

1605 Norwegian adolescents were followed from early (baseline, mean age 14.2 years) to late adolescence (follow-up, mean age 18.2 years). Maturational timing was assessed by self-reports of pubertal status (PDS) in boys and age at menarche (AAM) in girls. Central adiposity was classified according to waist circumference (waist) measured at baseline, using age and gender specific medians as cut off. Overweight was classified according to International Obesity Task Force (IOTF) standards.

**Results:**

At follow-up, early sexual maturation in girls, but not in boys, was associated with overweight. This association, however, was restricted to girls with high waist circumference (> median) at baseline (OR, 2.7, 95% CI 1.5–4.9). Thus, age at menarche was not associated with overweight in girls with low waist (≤ median) at baseline. Central adiposity was, independent of maturational timing, associated with higher BMI at follow-up in both genders, but differences were more pronounced among early matured girls (3.5 kg/m^2^), than among intermediate (2.7 kg/m^2^) and late matured girls (1.2 kg/m^2^).

**Conclusion:**

In girls, the combination of central adiposity and early age at menarche appears to increase the risk of being overweight in late adolescence.

## Background

The global increase in overweight and obesity at a young age [1] has alarming prospects, due to its long-term association with the risk of cardiovascular disease and diabetes as well as other chronic diseases [2,3]. Since the amount of body fat increases secondary to pubertal development, adolescence may be a critical period for developing overweight [4].

Studies have shown that those who mature early or more rapidly have higher body-mass index (BMI) and are at greater risk of developing overweight in adolescence and also in adulthood [5-9]. Those who mature early also tend to have more of their subcutaneous fat on the trunk than same aged others [6,10]. The latter observation may be particularly important because central fat may be a greater threat to health than peripheral fat [11].

The biological mechanisms underlying the association between early maturation and overweight are still unclear, but since overweight children tend to undergo their sexual maturation earlier than leaner children [12], subsequent overweight has been attributed to over-nutrition in childhood [13-15]. The joint effect of early sexual maturation and early adiposity may be central for the development of overweight [13,15]. Others have suggested that the association between early sexual maturation and adult obesity is independent of nutritional status at an early age [8]. It is, however, difficult to isolate the effects of early adiposity from the effects of early maturation. One common problem has been the reliance on body-mass index (BMI) as indicator of body fatness, which in young people may reflect maturational status as well as fatness [16,17]. Overweight is therefore suggested to be overestimated among those who mature early [13].

The aim of this study was to examine whether the association between the timing of sexual maturation and subsequent fatness (i.e. BMI and overweight) in late adolescence was modified by boys' and girls' level of central adiposity (i.e. waist circumference) as assessed in early adolescence.

## Methods

### Materials

This is a prospective study of 1605 adolescent students (the Young-HUNT Study) in Nord-Trøndelag County in Norway, who was examined twice with four years apart. The baseline study was conducted in middle school (age 12–16 years) between August 1995 and June 1997 (baseline). The Young HUNT 2 study (follow-up) took place between January 2000 and June 2001, when the students were in 12^th ^or 13^th ^grade in high school (age 16–20 years). The overall attendance was 95% at baseline and 86% at follow-up. The baseline and follow-up study were performed with identical and standardized procedures. Within a month after completing a self-administered questionnaire the students underwent clinical examinations, including measurements of height, weight, and waist and hip circumference. Among the 1605 participants with clinical assessments, 697 boys and 864 girls with registered information about maturational timing were eligible for analyses.

### Measurements

Self-reports of pubertal status in boys and age at menarche in girls were used to indicate timing of sexual maturation. We classified each individual as early or late relative to intermediate maturing boys and girls of the same age. At baseline, boys were asked to assess changes in secondary sexual characteristics by use of the Pubertal Developmental Scale (PDS). The PDS was originally developed by Petersen *et al*, and has later been validated [18,19]. In our study, reliability tests showed good internal consistency between the reported characteristics of sexual maturation (α = 0.85). The boys rated themselves according to growth spurt and pubic hair growth; and were asked to assess changes in voice and facial hair growth. On a scale ranging from 1 (have not begun) to 4 (development completed) they were asked to report the extent of pubertal changes. Total score for the four items was summarized and divided by 4, in order to maintain the original range from 1 to 4. Stratified by age, we classified boys who had PDS score in the lowest quintile as "late" matured, and those who were in the highest quintile as "early" matured. Those who scored between these extremes were classified as "intermediate" (Table 1).

**Table 1:** Characteristics of the study cohort at baseline

		Timing of sexual maturation		
		
Characteristics	Gender	Early	Intermediate	Late
Age, mean (95% CI)	boys	14.3 (14.2–14.5)	14.2 (14.1–14.3)	14.0 (13.9–14.1)
	girls	14.2 (14.1–14.3)	14.3 (14.2–14.4)	14.2 (14.1–14.3)
Pubertal status (PDS), mean score (95% CI)^a^	boys	3.1 (3.1–3.2)	2.4 (2.4–2.4)	1.7 (1.6–1.7)
Age at menarche, mean years (95% CI)	girls	11.7 (11.6–11.8)	13.1 (13.0–13.1)	14.8 (14.7–14.9)
Waist circumference, mean ^a ^(95% CI)	boys	73.6 (72.4–74.7)	72.1 (71.3–72.8)	69.8 (68.7–70.9)
	girls	68.4 (67.4–69.5)	68.6 (67.9–69.2)	65.8 (64.8–66.8)
Body mass index, mean ^a ^(95% CI)	boys	20.7 (20.3–21.1)	19.9 (19.6–20.2)	19.2 (18.8–19.7)
	girls	21.1 (20.7–21.5)	20.6 (20.3–20.8)	18.9 (18.5–19.3)

^a ^Age-adjusted means

At baseline, girls were asked to report their age at menarche (AAM) in years and months. Reliability of self-reported AAM is well established [20]. One fifth of the responders had not yet reached menarche (n = 158), or had incomplete or missing data (n = 30). For these girls we relied on information on AAM collected at follow-up. Mean AAM was 13.2 years (SD = 1.2). We classified girls whose menarche occurred before 12.5 years (20%) as "early" matured, girls with AAM between 12.5–13.9 years (60%) were classified as "intermediate", and girls with AAM at 14.0 years or later (20%) as "late" (Table 1).

Anthropometry was measured with the participants wearing light clothes and no shoes. Height and waist circumference was read to the nearest centimetre (cm) and weight to the nearest half kilogram (kg). We used waist circumference, as assessed at baseline as indicator of level of central adiposity (Table 1). Participants with waist circumference above the median within each gender and age group (i.e. half-year groups) were classified as having "high waist" and those below or at the median, were classified as having "low waist". Validation studies have shown high correlation between waist circumference and trunk fat measured by DEXA- scan, that measures both fat and lean body mass, among 3–19 years old children and adolescents [21,22].

As outcome, we used BMI and overweight as registered at follow-up in late adolescence. Body-mass index (BMI) was calculated as weight in kilograms divided by the squared value of height in meters (kg/m^2^). Overweight was classified according to The International Obesity Task Force (IOTF) standards, which link BMI percentiles during childhood and adolescence to the widely used adult cut-off-points of 25 kg/m^2 ^at age 18, as a definition of overweight [23].

### Ethics

Participation was voluntary and each student signed a written statement of consent. For students younger than 16 years of age, we also obtained written consent from their parents. The Young HUNT study was approved by the Regional committee for ethics in medical research, and by the Norwegian Data Inspectorate.

### Statistics

We examined the independent and modifying effect of early central adiposity (i.e. waist circumference > median) on the association between timing of sexual maturation and subsequent fatness (i.e. overweight and BMI) by multivariate logistic analyses and linear regression analyses, and by stratification. Logarithmic (ln) transformation of BMI at follow-up was used to fit model assumptions in linear regression analyses. Logistic regression analysis was used to study the association between timing of sexual maturation and prevalence of overweight at follow-up, in groups characterised by high (> median) or low (≤ median) waist circumference at baseline, adjusting for potential confounding of age, waist circumference, parental education level, frequency of leisure time physical activity, and frequency of intake of sweets and soft drinks as reported at baseline. We used a general linear model to compute multivariate adjusted mean BMI and mean differences in BMI at follow-up, conducted separately for early, intermediate and late matured boys and girls, adjusting for the potential confounding of age, parental education level, frequency of leisure time physical activity and of intake of sweets and soft drinks as reported at baseline. Precision of the estimates was assessed with 95% confidence intervals (CI) and all reported *P*-values are two-sided. All statistical analyses were performed using SPSS for Windows, version 13 (SPSS, Chicago, USA).

## Results

### Baseline characteristics

At baseline, pubertal status in boys and age at menarche in girls differed significantly between individuals classified as early, intermediate and late (Table 1). Age adjusted mean waist circumference (Table 1) was higher in early (73.6 cm) maturing boys, compared to boys who were intermediate (72.1 cm) (*P-value *= .03) or late matured (69.8 cm) (*P-value *< .001). Waist circumference did not differ significantly between early (68.4 cm) and intermediate (68.6 cm) maturing girls, whereas late maturing girls on average were leaner (65.8 cm) (*P-value *< .001) than intermediate matured girls. Age adjusted mean BMI at baseline differed significantly between all timing categories and was highest among early maturing boys (20.7 kg/m^2^) and girls (21.1 kg/m^2^) and lowest among late maturing boys (19.2 kg/m^2^) and girls (18.9 kg/m^2^).

### Tracking of adiposity from baseline to follow-up

During follow-up, mean BMI increased by more than 2 kg/m^2 ^and in late adolescence, overweight was more common in all timing categories than at baseline, except for boys with late sexual maturation. Among those classified as overweight at baseline, 80% of boys and 70% of girls were also classified as overweight at follow-up (data not shown).

### The prevalence of overweight at follow up

At follow-up, 20% of boys and 19% of girls were classified as overweight. Overweight was more than twice as common among early matured boys and girls (26%), compared to boys (11%) and girls (10%) who had matured late. After adjustment for age, parental education level, frequency of leisure time physical activity and frequency of intake of sweets and soft drinks as reported at baseline, early maturing girls, but not boys, were slightly more likely (OR, 1.6, 95% CI 1.0 -2.5) to be overweight, compared to girls who were intermediate matured (*P-value *= .03). Late matured boys (OR, 0.5, 95% CI 0.3–0.9) and girls (OR, 0.5, 95% CI 0.3–0.8) were both less likely to be overweight compared to intermediate matured counterparts.

### The modifying influence of waist circumference

We studied whether the association between timing of sexual maturation and overweight at follow-up could be modified by level of central adiposity at baseline (indicated by waist circumference). The results showed that for girls, but not for boys, there was a statistically significant interaction between timing categories and baseline waist circumference (*P-value *= .006). Further stratified analysis showed that among girls with low waist circumference (≤ median), early AAM was not associated with subsequent overweight at follow-up (Table 2). Among girls with high waist (> median), early matured girls were 2.7 times as likely (95% CI 1.5–4.9) to be overweight, compared to intermediate matured girls within this group. Odds ratios were adjusted for age, waist circumference, parental education level, physical activity and intake of soft drinks and sweets as obtained at baseline (Table 2).

**Table 2:** Odds ratio (OR) of being classified as overweight a at follow-up in late adolescence associated with timing of sexual maturation stratified by level of central adiposity (i.e. waist circumference)

	High waist circumference (> median)					Low waist circumference (≤ median)				
	
Timing	Overweight^a^	Normal weight	OR^b^	OR^c^	95% CI	Overweight^a^	Normal weight	OR^b^	OR^c^	95% CI
**Boys**										
Early	37	66	0.9	1.0	0.6–1.9	7	56	2.4	0.9	0.2–4.2
Intermediate	62	105	1.0	1.0	Reference	9	172	1.0	1.0	Reference
Late	19	35	0.9	0.8	0.3–1.7	0	110	NC	NC	-
**Girls**										
Early	42	50	1.8	2.7	1.5–4.9	2	81	0.5	0.5	0.1–2.7
Intermediate	78	164	1.0	1.0	Reference	11	220	1.0	1.0	Reference
Late	10	52	0.4	0.3	0.1–0.9	8	115	1.4	2.2	0.8–6.2

^a ^Classified according to the International Obesity Task Force (IOTF) percentile cut-off points for children and adolescents, ^b ^Unadjusted OR, ^c^Adjusted for baseline age, waist circumference, dietary sugar intake, leisure time physical activity and parental education level, CI denotes confidence interval and NC denotes not calculated

By use of linear regression analyses, we estimated the independent and combined influence of timing of sexual maturation and waist circumference on mean BMI at follow-up. In boys (Table 3), both pubertal status score (*P-value *= .005) and waist circumference (*P-value *< .000) at baseline (age-adjusted), contributed independently to BMI at follow-up (Model 1), but the interaction term was not statistically significant (Model 2). In girls, age at menarche (*P-value *= .002) and waist circumference (*P-value *< .000) were both independently related to BMI (Model 1), but in girls these effects were interactive (Model 2, *P-value *= .002). Inclusion of the interaction term, however, did not change the adjusted R^2 ^(Table 3).

**Table 3:** The independent and combined influence of timing of sexual maturation (SM timing^a^) and waist circumference on log-transformed mean BMI at follow-up, reported as standardised beta coefficients^b^

Independent variables	*Boys*				*Girls*			
	
	Model 1		Model 2		Model 1		Model 2	
	
	β	*P*-value	β	*P*-value	β	*P*-value	β	*P*-value
SM timing^a^	0.09	< .000	0.12	.65	-0.09	.002	0.68	.006
Waist circumference	0.68	.005	0.69	< .000	0.60	< .000	1.53	< .000
SM timing*waist circumference			-0.03	.92			-1.11	.002
*Model, adjusted R^2^*	*0.48*		*0.48*		*0.39*		*0.39*	

^a ^SM timing denotes pubertal status in boys and age at menarche in girls, both included as continuous variables in analyses

^b ^Coefficients are adjusted for age at baseline.

Further stratified analyses showed that across all maturational timing groups, mean BMI at follow-up was significantly higher among boys and girls with high waist circumference (> median), compared to those with low circumference (≤ median) at baseline (Table 4). In girls, however, differences appeared to be more pronounced among those with early age at menarche (3.5 kg/m^2^, 95% CI 1.7–5.3) than among intermediate (2.7 kg/m^2^, 95% CI 1.8–3.6) and late matured girls (1.2 kg/m^2^, 95% CI 0.1–2.2). Differences in age and height adjusted weight at follow-up, showed that early matured girls with high waist circumference at baseline (> median) weighed on average 11.2 kg more than those with low waist (≤ median) (*P-value *< .001) (data not shown).

**Table 4:** Mean BMI (kg/m^2^) at follow-up related to level of central adiposity (i.e. waist circumference) at baseline, stratified by timing of sexual maturation (SM timing) and gender

	High waist circumference (> median)		Low waist circumference (≤ median)			
			
SM timing	No. of participants	Mean ^a ^BMI (95% CI)	No. of participants	Mean ^a ^BMI (95% CI)	Mean^a ^difference(95% CI)	P-value
*Boys*						
Early	103	24.7 (23.7–25.6)	60	21.8 (20.7–22.9)	2.9 (1.4–4.4)	< .001
Intermediate	167	24.9 (24.1–25.7)	180	21.5 (20.7–22.3)	3.4 (2.3–4.5)	< .001
Late	52	23.8 (22.5–25.1)	109	20.5 (19.5–21.5)	3.3 (1.6–4.9)	< .001
*Girls*						
Early	89	24.7 (23.4–25.9)	83	21.2 (19.9–22.4)	3.5 (1.7–5.3)	< .001
Intermediate	236	24.0 (23.4–24.6)	224	21.3 (20.7–22.0)	2.7 (1.8–3.6)	< .001
Late	58	22.2 (21.3–23.0)	121	21.0 (20.3–21.7)	1.2 (0.1–2.2)	.04

^a ^Means, adjusted for age at follow-up and dietary sugar intake, leisure time physical activity and parental education reported at baseline, CI denotes confidence interval

## Discussion

In this prospective study of boys and girls we found that early age at menarche in girls was associated with overweight in late adolescence, but the association was only present in girls with relatively high central adiposity at baseline (waist circumference above the median).

Longitudinal studies have shown that differences in BMI and body fatness related to sexual maturation may persist, also after the completion of biological growth and development [7,10,24-26]. Some previous cross-sectional studies have reported a relatively strong association between early sexual maturation and overweight in girls [5,8,9], but limited research has been conducted in boys. Since sexual maturation tends to occur earlier in overweight children, it has been suggested that early adiposity may play an important role for subsequent fatness. However, it is not easy to single out the independent influence of early sexual maturation on this association. In women, it was recently reported that nearly all the influence of early menarche on adult overweight could be attributed to childhood adiposity [15]. Other studies have shown that the influence of early age at menarche on adult obesity could be mediated by childhood adiposity, but that the whole effect could not be explained by this [13,14]. In our study, we found that early menarche was not associated with overweight in girls that were relatively lean at the entry of the study, i.e. in early adolescence.

Many factors may contribute to early menarche, and genetic make up is probably influential for the timing of all pubertal milestones. The onset of puberty may also represent a sensitive early marker of interactions between environmental conditions and genetic susceptibility [27]. The single most important environmental factor is childhood over-nutrition that may have profound effects on the tempo of growth and puberty [28,29]. Thus, the influence of childhood adiposity on subsequent timing of puberty has been observed in very young children [12,30,31]. Given that the timing of puberty is triggered by childhood over-nutrition, obese children who mature early may differ from other early maturing children in many respects. In another study conducted on this population, we found that early maturation combined with early central adiposity was associated with normal final height, while early maturation combined with "leanness" at a young age was associated with relatively short final stature [32]. These results suggest that differences in stature and fatness in late adolescence that could be linked to early central adiposity were more pronounced among those who matured early compared to those who matured later, and this pattern was especially apparent in girls. Previous studies have reported a joint effect between childhood BMI and age at menarche on adult BMI [13,15], but the heterogeneity in effect related to early sexual maturation has to our knowledge not previously been described.

Adolescence may be a critical period for developing overweight [4], but results have also indicated that the major part of early adiposity may be fully developed by 11 years of age, or earlier [33]. Although we found that the overall prevalence of overweight increased gradually from early to late adolescence, nearly all participants who were overweight at follow-up had central adiposity at baseline. Among those with central adiposity above the median, however, early maturing girls were nearly three times as likely to be classified as overweight at follow-up as intermediate matured girls.

Even if waist circumference percentile charts have been described [34], appropriate cut-off points for defining overweight have not yet been identified. Compared to British adolescents for example, participants in this study (1995–97) were on average "leaner" than adolescents in a British study from 1997, but "fatter" than the British were before 1990 [34]. This may reflect country specific increases in adolescent body size over the last few decades. About half the girls with early age at menarche in our study were relatively lean at baseline (indicated by waist circumference) and remained lean at follow-up. This appears to be in contrast to the findings of Must et al, who found that the leanest girls had not experienced early age at menarche [15]. In that study, however, the definition of leanness was more stringent, and early adiposity was accounted for by the use of BMI and not waist circumference, as in our study.

Possible health risks associated with high waist circumference may not be clear, but this measure has been recommended in studies of children and adolescents [1]. Studies have shown that waist circumference has increased in children and adolescents since the 1990s, and appears to exceed the corresponding increase in BMI [34]. Compared to the use of BMI, waist circumference has shown to be less affected by gender, race and pubertal maturation [21,35]. In our study, mean waist circumference did not differ significantly between early and intermediate matured girls at baseline, but was slightly lower among late maturing girls. Early maturing boys, however, were less likely to be classified as lean and thus, central adiposity may be overestimated in this group. Our study revealed quite different results for boys and girls, especially in relation to central adiposity. Due to the gender dimorphism in fat deposition [21] and since the typical adult fat distribution is gained at an older age in boys than in girls [36], gender comparisons may not be appropriate. Different indicators of sexual maturation were also considered for boys and girls and could explain some of the discrepancies in findings. Others, however, have also reported gender differences related to early sexual maturation and overweight [9], which may involve both genetic as well as endocrine factors.

There are several limitations associated with the use of self-reports of pubertal events. Short-term-recalls of AAM, however, is considered reliable [20], and has been extensively used in epidemiological studies. Validation studies have also shown moderate to good agreement between self-evaluations and physicians' ratings of sexual maturation, but ratings may be overestimated in early stages of puberty and in obese children [19,37-39]. In our data there was high consistency between self-reports of pubertal status and clinically obtained height and weight measurements at baseline. Another limitation was that we had no access to childhood growth data and thus, we were not able to study the possible link between childhood over-nutrition, age at onset of puberty and subsequent fatness during adolescence.

Main strengths of our study include the longitudinal design, its high attendance, and the use of standardised anthropometric measurements. Due to Caucasian homogeneity, ethnic origin is unlikely to explain the observed variation in maturation and body composition in this population.

## Conclusion

We found that the increased risk of early maturing girls to be overweight in late adolescence was confined to girls with relatively high central adiposity in early adolescence. Thus, the combination of early adiposity and early age at menarche appears to increase the risk of being overweight in late adolescence.

## Competing interests

The author(s) declare that they have no competing interests.

## Authors' contributions

GHB and LJV contributed significantly in all parts of the research process. TLH was responsible for the data collection and administration of the Young HUNT study. TILN contributed in statistical analysis and in interpretation of results. All co-authors critically reviewed the process and provided suggestions for manuscript revisions. They have all read and approved the final manuscript.

## Pre-publication history

The pre-publication history for this paper can be accessed here:


